# A Meta-Analysis of Human Transcriptomics Data in the Context of Peritoneal Dialysis Identifies Novel Receptor-Ligand Interactions as Potential Therapeutic Targets

**DOI:** 10.3390/ijms222413277

**Published:** 2021-12-10

**Authors:** Michail Evgeniou, Juan Manuel Sacnun, Klaus Kratochwill, Paul Perco

**Affiliations:** 1 Division of Pediatric Nephrology and Gastroenterology, Department of Pediatrics and Adolescent Medicine, Comprehensive Center for Pediatrics, Medical University Vienna, Währinger Gürtel 18-20, 1090 Vienna, Austria; michail.evgeniou@meduniwien.ac.at (M.E.); n11927077@students.meduniwien.ac.at (J.M.S.); klaus.kratochwill@meduniwien.ac.at (K.K.); 2 Christian Doppler Laboratory for Molecular Stress Research in Peritoneal Dialysis, Department of Pediatrics and Adolescent Medicine, Medical University Vienna, Währinger Gürtel 18-20, 1090 Vienna, Austria; 3 Zytoprotec GmbH, 1090 Vienna, Austria; 4 Department of Internal Medicine IV, Medical University Innsbruck, Anichstrasse 35, 6020 Innsbruck, Austria

**Keywords:** peritoneal dialysis, transcriptomics, meta-analysis, mesothelial cells, receptor-ligand interactions

## Abstract

Peritoneal dialysis (PD) is one therapeutic option for patients with end-stage kidney disease (ESKD). Molecular profiling of samples from PD patients using different Omics technologies has led to the discovery of dysregulated molecular processes due to PD treatment in recent years. In particular, a number of transcriptomics (TX) datasets are currently available in the public domain in the context of PD. We set out to perform a meta-analysis of TX datasets to identify dysregulated receptor-ligand interactions in the context of PD-associated complications. We consolidated transcriptomics profiles from twelve untargeted genome-wide gene expression studies focusing on human cell cultures or samples from human PD patients. Gene set enrichment analysis was used to identify enriched biological processes. Receptor-ligand interactions were identified using data from CellPhoneDB. We identified 2591 unique differentially expressed genes in the twelve PD studies. Key enriched biological processes included angiogenesis, cell adhesion, extracellular matrix organization, and inflammatory response. We identified 70 receptor-ligand interaction pairs, with both interaction partners being dysregulated on the transcriptional level in one of the investigated tissues in the context of PD. Novel receptor-ligand interactions without prior annotation in the context of PD included BMPR2-GDF6, FZD4-WNT7B, ACKR2-CCL2, or the binding of EPGN and EREG to the EGFR, as well as the binding of SEMA6D to the receptors KDR and TYROBP. In summary, we have consolidated human transcriptomics datasets from twelve studies in the context of PD and identified sets of novel receptor-ligand pairs being dysregulated in the context of PD that warrant investigation in future functional studies.

## 1. Introduction

Peritoneal dialysis is one therapeutic option next to hemodialysis and renal transplantation for patients with end-stage kidney disease. A number of complications such as angiogenesis, inflammation, or fibrotic processes, however, hamper long-term success of this form of dialysis, with a substantial number of patients needing to switch to hemodialysis over time [1]. PD-associated complications are to a large extent driven by the currently used PD fluids, namely, their high concentration of glucose, their low pH, and their high content of toxic glucose degradation products [2].

High-throughput omics technologies such as microarrays, RNA-sequencing, or proteomics methods allow for simultaneous measurements of thousands of molecular features and support elucidation of dysregulated processes in diseases along with the identification of molecular biomarkers and novel drug targets. A combined analysis of transcriptomics and proteomics data in the context of PD recently led to the identification of the beneficial impact of lithium on peritoneal membrane integrity, thus demonstrating that an integrated analysis of Omics data can also lead to novel therapeutic strategies [3]. A number of individual Omics studies in the context of PD have been published in the past couple of years investigating the impact of different dialysis fluids or different additives on patient outcome and the discovery of associated molecular pathways [4,5]. In particular, gene expression profiling has been used to characterize changes on the transcriptional level in different cell types such as mesothelial cells, peritoneal cells, and endothelial cells, as well as in peripheral blood mononuclear cells (PBMCs) playing a major role in inflammatory responses linked to PD-associated complications.

The aim of the present study was to consolidate available transcriptomics profiles in the context of peritoneal dialysis to study dysregulated molecular pathways with a specific focus on differentially expressed receptor-ligand pairs and receptor-receptor complexes, which could serve as novel therapeutic targets to counterbalance peritoneal dialysis associated complications.

## 2. Results and Discussion

### 2.1. Transcriptomics Studies Included in Our Meta-Analysis

We identified thirteen TX studies in the context of PD that fulfilled our inclusion criteria, namely, being untargeted TX studies in human samples. Twelve were finally included into our meta-analysis as we were not able to get access to either the raw data or the full list of differentially expressed genes for one study. These 12 studies were published between 2008 and 2020, with five of the twelve studies (42%) being published within the last three years. Six studies (50%) focused on expression changes in mesothelial cells, three studies (25%) investigated expression profiles in peritoneal cells, two studies focused on PBMCs (17%), and one study (8%) investigated expression changes in omental arterioles (OA). A detailed listing of the 12 included studies is given in Table 1 with information on the used assay platform, sample type, and the different groups that have been investigated, along with references to the original studies. Despite the fact that the individual study setups were heterogeneous across the twelve studies, for each study, we were able to define a case group showing a more advanced stage of disease or a higher degree of damage as compared with a respective control group. In some studies, comparisons have, for example, been conducted between samples from PD patients as compared to samples from patients with chronic kidney disease not yet on dialysis. Other studies have investigated the impact of optimized PD fluids on gene expression as compared with control medium, with one study investigating the differences in gene expression patterns when comparing peritoneal cells from long-term PD patients versus short-term PD patients.

### 2.2. Differentially Expressed Genes

**Figure 1:**
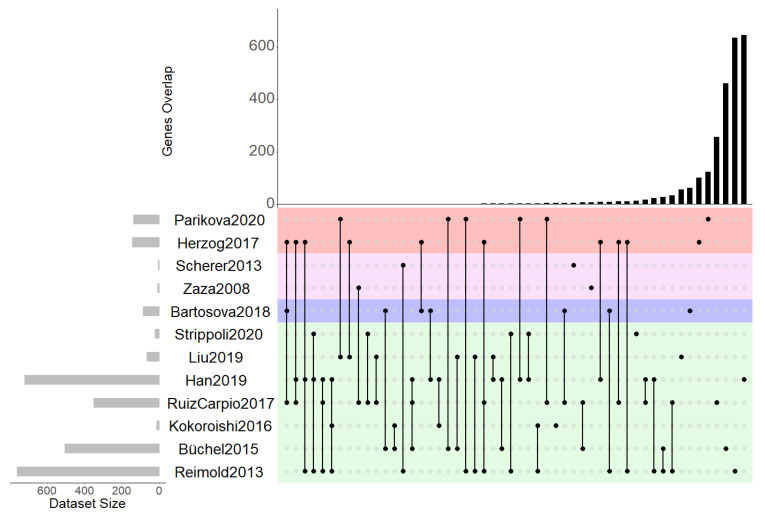
UpsetR plot of differentially expressed genes indicating overlapping genes across the individual studies. Color-coding indicates the four different sample types, namely, mesothelial cells (green), omental arterioles (violet), PBMCs (pink), and peritoneal cells (red).

We identified 3179 differentially expressed transcripts in total that were mapped to 2591 unique differentially expressed genes (DEGs), and 1360 genes were reported to be upregulated in the diseased/damaged state as compared with the control group, with 1346 genes showing downregulation in the diseased/damaged state. The contribution of DEGs from the individual studies to the final set of DEGs varied substantially, ranging from 7 DEGs from the study by Scherer (2013) [8] to 761 DEGs being reported in study by Reimold (2013) [7]. When analyzing the lists of DEGs in the context of the different sample types studied, the largest set of DEGs was available for mesothelial cells (*n* = 2286), followed by peritoneal cells (*n* = 282), omental arterioles (*n* = 85), and PBMCs (*n* = 16). Figure 1 shows the overlap of DEGs across the individual studies. Two-hundred and twenty DEGs have been found in at least two studies, with fibrillin 1 (FBN1) being reported to be downregulated in three studies and across two different tissues, namely, mesothelial cells and peritoneal cells. FOSB (FosB proto-oncogene, AP-1 transcription factor subunit) was found to be upregulated in mesothelial cells in three independent studies. CFH (complement factor H) was reported to be differentially regulated in three different studies showing upregulation in mesothelial cells and omental arterioles and downregulation in peritoneal cells.

Upregulated ligands supported by multiple studies included CCL2 (C-C motif chemokine ligand 2), COL16A1 (collagen type XVI alpha chain 1), COL1A2 (collagen type I alpha chain 2), COL3A1 (collagen type III alpha chain 1), or fibronectin (FN1) with thrombospondin (THBS1), SPP1 (secreted phosphoprotein 1), COL6A3 (collagen type VI alpha chain 3), and FBN1 (fibrillin), which were found to be consistently downregulated across different studies.

Interestingly, some receptors such as the epidermal growth factor receptor (EGFR) have been found to be significantly up- as well as downregulated in mesothelial cells in the more damaged state as compared with the control state.

### 2.3. Functional Analysis—Gene Set Enrichment Analysis

In order to evaluate which biological processes and mechanisms are affected in the context of PD, we performed gene set enrichment analysis using the full set of DEGs but also the sample type specific DEG subsets for mesothelial cells, omental arterioles, peritoneal cells, and PBMCs, respectively. We identified 41 enriched gene ontology (GO) biological processes that showed statistically significant enrichment after correction for multiple testing based on at least one of the five input sets of DEGs. Angiogenesis was the most significant term based on the full set of DEGs, followed by cell adhesion, cell division, and cell migration. The full listing of enriched GO biological processes is given in Table 2. Inflammatory response was the only GO biological process term found to be significantly enriched based on the limited set of only thirteen DEGs in PBMCs. There was a strong overlap in enriched GO biological processes between the full set of DEGs and the set of DEGs found in mesothelial cells potentially caused by the fact that the set of mesothelial DEGs made up ~88% of the full set of DEGs. In peritoneal cells, cell adhesion and extracellular matrix organization were enriched, whereas in omental arterioles the alternative pathway of complement activation and Rho protein signal transduction were enriched.

### 2.4. Receptor-Ligand Interaction Analysis

**Figure 2:**
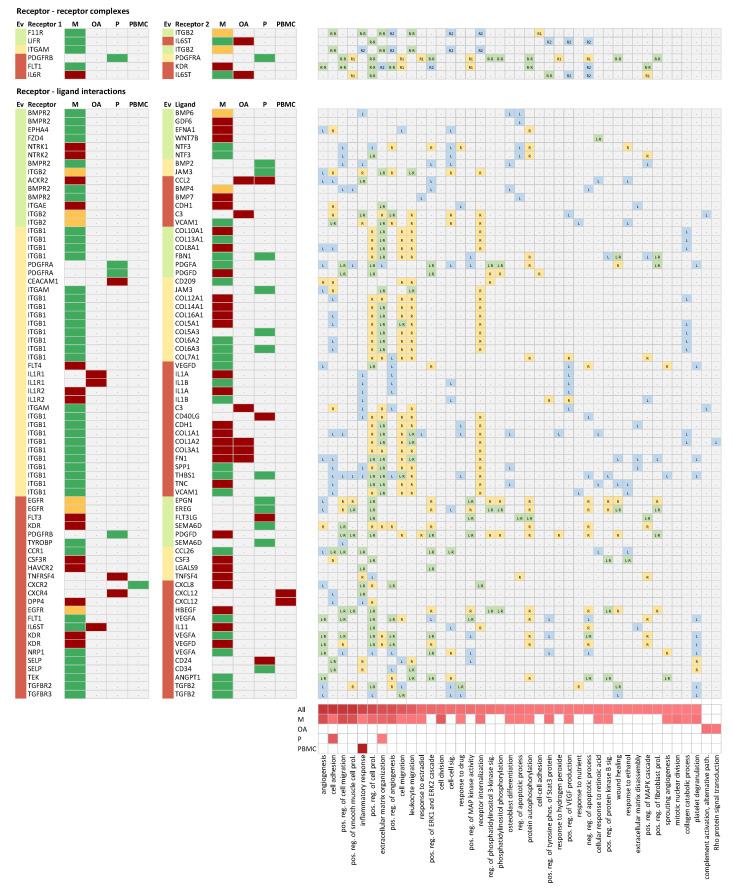
Receptor-receptor complexes and receptor-ligand interactions. Each row holds one receptor-receptor complex or receptor-ligand interaction, with both interactors being differentially expressed in at least one sample type in the context of PD. The evidence category (Ev) is color-coded for each protein depending on whether there is no literature information (green), some literature information (orange), or abundant literature information available in the context of PD, as described in the Section 3. The columns M (mesothelial cells), OA (omental arterioles), P (peritoneal cells), and PBMC (peripheral blood mononuclear cells) indicate regulation of transcripts in the respective samples type, with red indicating upregulation, green indicating downregulation, and orange indicating up- and downregulation, in different studies in the same samples type. The heatmap on the right indicates which of the two interactors is linked to the respective enriched GO biological process. Green indicates that both interactors are assigned to the respective GO biological process, yellow indicates that the receptor 1 is assigned to the respective GO biological process, and blue indicates that the ligand or the receptor 2 is assigned to the respective GO biological process. phosp. = phosphorylation; pos. = positive; reg. = regulation; path. = pathway; sig. = signaling; and prol. = proliferation.

Next, we were interested in receptor-ligand interactions as well as receptor-receptor complexes, with both interactors showing differential expression on the transcript level in the consolidated set of DEGs. We extracted receptor-ligand interaction pairs and receptor-receptor complexes from CellPhoneDB version 2.0 [16]. In our analysis, we focused on the subset of 878 experimentally verified interactions and complexes. We identified six unique receptor-receptor complexes made up of 10 unique genes, with both receptors being among the set of DEGs. In addition, we identified 70 unique receptor-ligand interactions, with both interactors being dysregulated in the context of PD. This set of receptor-ligand interactions was made up of 34 unique receptors and 56 unique ligands. Sixty-three of these receptor-ligand pairs were linked to at least one of the enriched GO biological processes, with either the receptor and/or the ligand being associated with the respective GO biological process as depicted in Figure 2. The biological process associated with the most receptor-ligand pairs was extracellular matrix organization driven by both ITGB1 (integrin subunit beta 1) and ITGB2 (integrin subunit beta 2) and being in the list of cellular-receptor-forming associations with a number of secretory ligands, among them a number of collagen fragments.

Among the receptor-ligand pairs, we found a few well-studied interactions such as the links between TGFB2 (transforming growth factor beta 2) and its two receptors TGFBR2 (transforming growth factor beta receptor 2) and TGFBR3 (transforming growth factor beta receptor 3), and the binding of certain isoforms of the vascular endothelial growth factor (VEGFA and VEGFD) to the kinase insert domain receptor (KDR), fms-related receptor tyrosine kinase 1 (FLT1), and neuropilin 1 (NRP1) in the context of angiogenesis. Receptor-ligand interactions linked to inflammation included C-X-C motif chemokine ligand 12 (CXCL12)-C-X-C motif chemokine receptor 4 (CXCR4) or CXCL12-dipeptidyl peptidase 4 (DPP4). Interleukin 11 (IL11) was the highest upregulated ligand binding to the interleukin 6 signal transducer (IL6ST) involved in cell-cell signaling. All these interactions were classified into evidence category three (indicated by the light red color-code in Figure 2 in the Ev column), i.e., being already discussed in scientific literature in the context of PD.

We identified a number of interesting interactions that have not yet been reported in the context of PD, based on literature evidence that we tagged as evidence level one (green color-code in Figure 2 in the Ev column). The most relevant ones being associated with enriched molecular pathways in the context of PD will be discussed in the following subsections.

#### 2.4.1. Receptor-Receptor Complex between Leukemia Inhibitory Factor Receptor (LIFR) and IL6ST

LIFR is a type I cytokine receptor that forms a complex with IL6ST. IL6ST is also known as CD130 or GP130 (glycoprotein 130) and is involved in processes of cell proliferation, differentiation, and survival. Both receptors were found to be strongly downregulated on the transcriptional level in mesothelial cells. LIFR showed a fold-change of −11 and IL6ST showed a fold-change of −5.9, respectively. Interestingly, IL6ST was moderately upregulated (1.4-fold) in omental arterioles. Reduced serum levels of the leukemia inhibitory factor (LIF), one of the key ligands binding to this receptor complex, have been found to be associated with vasculopathy in patients with systemic sclerosis, together with downregulation of the two receptors, LIFR and IL6ST, in this patient group [17]. Reduced serum soluble GP130 (sGP130) levels were shown to be associated with coronary atherosclerosis severity [18]. Elevated sGP130 levels were in addition linked with reduced occurrences of myocardial infarction [19]. Diminished GP130 protein levels in the failing heart were reported by Zolk and colleagues, whereas the expression on the transcriptional level was enhanced at the same time [20]. Overall, there is evidence that the receptor complex LIFR-IL6ST is associated with cardiovascular disease, one of the key complications in peritoneal dialysis, making this receptor complex an interesting target for further investigation.

#### 2.4.2. Receptor-Ligand Interaction between the Atypical Chemokine Receptor 2 (ACKR2) and C-C motif Chemokine Ligand 2 (CCL2)

ACKR2 is a beta chemokine receptor that is expressed in a large number of tissues. CCL2, also known as monocyte chemoattractant protein 1 MCP-1, is a chemokine involved in inflammatory and immunoregulatory processes. ACKR2 was upregulated 5-fold in mesothelial cells with CCL2 being upregulated 2.5- and 1.6-fold in omental arterioles and peritoneal cells, respectively. Both proteins are linked to inflammation in cardiac tissue. ACKR2 was reported to prevent fibrosis in kidney tissue [21,22]. CCL2, on the other hand, was reported to induce fibrotic processes in renal tissue [23]. CCL2 has been suggested as a biomarker for monitoring inflammatory processes in the context of PD [24]. ACKR2 is a novel receptor that might be worth investigating in more detail in subsequent functional experiments in the context of inflammation in PD.

#### 2.4.3. Receptor-Ligand Interactions between Bone Morphogenic Protein Receptor Type 2 (BMPR2) and Growth Differentiation Factor 6 (GDF6) as Well as Bone Morphogenic Protein 6 (BMP6)

BMPR2 is a member of the BMP family of transmembrane protein kinases, forming an anchor for a number of ligands from the TGF-beta family of proteins. Two of these ligands are GDF6, also known as bone morphogenic protein 13 (BMP13), and BMP6. GDF6 was one of the ligands showing the strongest upregulation, with a fold-change of 11.9 in mesothelial cells. BMP6 was found to be up- and downregulated in mesothelial cells. BMPR2 itself was downregulated 6-fold in mesothelial cells. In general, crosstalk between transforming growth factor beta and BMP signaling cascades influence fibrosis in different tissues of relevance in the context of PD [25]. Compared to other ligands binding to this receptor, relatively little information is available for GDF6, although there is some evidence of its role in the context of renal dysfunction [26,27].

#### 2.4.4. Receptor-Ligand Interactions between the Epidermal Growth Factor Receptor (EGFR) and Epiregulin (EREG) as Well as the Epithelial Mitogen (EPGN)

EGFR modulates numerous molecular processes through homo- or hetero-dimerization with other tyrosine kinase receptors and has been described as potential pharmaceutical target for counterbalancing fibrotic processes in various tissues [28]. EGFR was found to be dysregulated in mesothelial cells, with one study reporting a 4.9-fold upregulation [12], whereas a second study reported EGFR to be downregulated by 4.6-fold in the diseases/damaged state [7]. EREG is a ligand binding to the EGFR, which is involved in cell proliferation and inflammation among other processes and was found to be 2-fold downregulated in peritoneal cells. EREG regulation is associated with progression in various tumor types [29,30,31] but was also reported to play a role in the progression of pathological conditions in cardiac tissue in the context of myocardial infarction [32]. EPGN, which was 1.7-fold downregulated in peritoneal cells, is the most recently discovered EGFR ligand [33]. Both EREG and EPGN belong to the low-affinity EGFR ligands that seem to be evolutionary related [34]. The associations with cardiovascular complications and the cross-talk between EREG and EGFR make also this interaction interesting in the context of PD.

#### 2.4.5. Receptor-Ligand Interaction between Frizzled Class Receptor 4 (FZD4) and Wnt Family Member 7B (WNT7B)

FZD4 is a receptor for ligands belonging to the family of WNT proteins that was downregulated 7.1-fold in mesothelial cells. WNT7B, one of the ligands of FZD4, on the other hand was upregulated 10.7-fold in mesothelial cells. Downstream of this receptor-ligand interaction, beta-catenin levels are regulated, in turn inducing leukocyte transendothelial migration in inflammatory and fibrotic processes [35]. Interfering with this receptor-ligand interaction to modulate inflammatory as well as fibrotic processes in PD patients warrants further investigation.

#### 2.4.6. Receptor-Ligand Interactions between KDR and Semaphorin 6D (SEMAD6) as Well as TYROBP and SEMAD6

KDR, also known as vascular endothelial growth factor receptor 2 (VEGFR2), is a type III receptor tyrosine kinase involved in inducing cell proliferation and processes related to cell migration and morphogenesis. In mesothelial cells we found a 3.7-fold upregulation, which is associated with fibrosis in PD patients. KDR was also found to be involved in CKD in general as well as in other indications such as liver disease [36,37,38]. SEMA6D is part of the semaphorin protein family that can act as ligand as well as receptor via reverse signaling [39,40]. SEMA6D is downregulated 1.7-fold in peritoneal cells. Next to its interaction with KDR, SEMA6D also binds to TYROBP (transmembrane immune signaling adaptor TYROB). This gene encodes a transmembrane signaling polypeptide containing an immunoreceptor tyrosine-based activation motif (ITAM) in its cytoplasmic domain to induce immune responses [41]. TYROBP was downregulated 6.5-fold in mesothelial cells. The link to angiogenesis and the immunomodulatory mechanisms of these receptor-ligand pairs also makes these interactions attractive for potential therapeutic interventions in the context of PD.

#### 2.4.7. Receptor-Receptor Complex between F11R and ITGB2

F11 receptor (F11R), also known as junctional adhesion molecule A (JAM-A), is a cell adhesion protein found in platelets. It is induced by inflammatory cytokines in endothelial cells and is associated with the onset of thrombotic and atherosclerotic events [42,43]. F11R was downregulated 5.3-fold in mesothelial cells. Integrin subunit beta 2 (ITGB2) is a member of the integrin family found to be involved in neutrophil activation, transmigration, or extravasation [44,45], which was found to be up− as well as down-regulated in mesothelial cells. F11R binds to ITGB2, thus facilitating transvasation of leukocytes through the endothelial barrier as well as platelet activation. This receptor complex formation correlates with atherosclerotic phenotype [46,47]. As previously stated, both infiltration of immune cell into the peritoneum as well as cardiovascular complications are well described in PD patients, making this pair a promising candidate for further studies.

#### 2.4.8. Receptor-Ligand Interaction between EPH Receptor A4 (EPHA4) and Ephrin A1 (EFNA1)

EPHA4 is an ephrin receptor that can bind—in contrast to other ephrin receptors—almost all ephrins, among them also EFNA1. EPHA4 was downregulated 3.1-fold, with its ligand EFNA1 being upregulated 2.9-fold in mesothelial cells. An association of EPHA4 with atrial remodeling, cardiomyopathy, stroke, and hypertension was shown in an EPHA4 knock-out mouse model [48]. Inhibition of EPHA4 was also found to decrease VEGF signaling, AKT signaling, and other angiogenic pathways, and its role as therapeutic target in different tumor entities was recently discussed [49]. Angiogenesis is a well-described mechanism in PD patients, being linked to poor cardiovascular outcome and loss of peritoneal barrier function, making this interaction interesting.

#### 2.4.9. Receptor-Ligand Interactions between NTRK1 and NTF3 as Well as NTRK2 and NTF3

Neurotrophic tyrosine receptor kinase 1 (NTRK1) is a member of the NTRK family, a group of tyrosine kinases. Due to some of the targets that NTRK1 phosphorylates, NTRK1 has been widely described as drug target in various tumors [50]. Neurotrophin 3 (NTF3), one of the NTRK1 ligands, along with other neurotrophins, is known for its neuroprotective capacity counterbalancing neural apoptosis [51]. Interestingly, in our data, both NTRK1 and NTRK2 receptors are upregulated 8.8- and 2.6-fold, with NTF3 being downregulated 2.3-fold in mesothelial cells. Upregulation of NTRKs has been previously linked to EMT in different cancer cell lines [52,53,54]. In PD, MMT is associated with loss of function of the peritoneal barrier. A pro-EMT environment due to upregulation of NTRKs in addition to the loss of the cytoprotective effect of NTF3 could possibly be linked to MMT in PD. Besides EMT, the neutrophin-NTRKs axis has also been found to play a role in different cardiopathies, both in the vasculature and in cardiac hypertrophy, being among the known complications in PD patients [55,56].

### 2.5. Study Limitations and Planned Next Steps

A limitation of our study combines only transcriptomic data—a decision made on the basis of the current data landscape—which is focused on identification of relevant receptor-ligand pairs that are driven by changes on the transcriptional level. We are thus not able to identify receptor-ligand interactions of interest that are regulated on the post-transcriptional level, e.g., by differential post-translational modification, translocation, or secretion. We furthermore restricted our analysis to those receptor-ligand pairs with both interactors being significantly differentially expressed. This strategy will yield false-negatives due to certain cellular signaling cascades that may be activated by increased abundance of only the ligand.

The individual studies in this analysis were very heterogeneous in their setup. Thus, it was not surprising that the overlap of differentially regulated genes was limited, with the individual studies providing complementary findings.

Functional verification of identified receptor-ligand interactions was beyond the scope of this exploratory hypothesis-generating meta-analysis. The consolidated dataset, however, may serve as a resource for researchers in the field of peritoneal dialysis to study their genes of interest along with the molecular neighborhood of those genes. The dataset also has the potential to support biomarker research, when looking for the highest up- or downregulated genes on the transcriptional level.

## 3. Materials and Methods

### 3.1. Defining the Set of Eligible Transcriptomics Studies in the Context of Peritoneal Dialysis for the Current Meta-Analysis

The current meta-analysis focused on untargeted human transcriptomics studies in the context of PD that used an approach. We therefore included all studies reporting results of whole-genome microarray or RNA sequencing experiments. We furthermore excluded studies solely focusing on non-protein coding RNAs (e.g., miRNAs) as well as studies with a focus on infections or malignancies (mesotheliomas).

We searched the PubMed database for PD transcriptomics datasets in December 2020 using the following search query: “(peritoneal dialysis OR mesothelial cell OR mesothelial cells) AND (gene expression profiling OR microarray)”. Titles and abstracts of resulting publications were screened for studies meeting inclusion and exclusion criteria as defined above. We subsequently searched the two gene expression repositories ArrayExpress and the Gene Expression Omnibus (GEO) using the following search queries: (“peritoneal dialysis” OR “mesothelial cell” OR “mesothelial cells”) AND (gene expression profiling OR microarray) NOT (mesothelioma OR cancer OR ovarian OR proteomic OR carcinoma OR melanoma). We further restricted the resulting list to studies in human.

We recorded the following information from studies considered for the current meta-analysis: (i) sample type, (ii) sample number, (iii) PubMed ID, (iv) GEO GSE code (if available), (v) experimental platform, (vi) conducted comparisons, and (vii) criteria de-fined by the authors to define differentially expressed genes.

### 3.2. Consolidating the Sets of Differentially Expressed Genes (DEGs)

For studies reporting and providing full lists of DEGs, we used those gene sets for our meta-analysis. The GEO2R tool was used to identify DEGs for studies not providing the full list of DEGs but having an entry in the GEO database. We used an FDR cutoff of <5%. The study by Büchel (2015) reported several comparisons that were averaged for significantly identified DEGs for further analyses [4].

The Ensembl Gene ID was used as common gene identifier and identified differentially expressed transcripts were mapped to the respective Ensembl Gene ID using Ensembl Biomart build “Ensembl Genes 103/GRCh38.p12”. The Ensembl Biomart was also subsequently used to annotate identified genes with official gene symbols and gene descriptions. For studies providing only gene symbols, we first mapped the provided gene symbols to the current official gene symbols using the alias2symbol function of the limma R package. The EdgeR R package was used to analyze the RNA sequencing dataset.

### 3.3. Functional Gene Set Enrichment Analysis

The Database for Annotation, Visualization, and Integrated Discovery (DAVID) tool V6.8 was used to identify enriched gene ontology (GO) biological processes [57]. We used the full set of unique DEGs as input set of the gene set enrichment analysis as well as sample-type-specific DEG subsets for mesothelial cells, omental arterioles, peritoneal cells, and PBMCs, respectively.

### 3.4. Receptor-Ligand Interactome Analysis

We extracted data from CellPhoneDB version 2.0 on receptor-receptor complexes and receptor-ligand interactions in order to identify novel receptor-ligand interactions showing differential expression in PD [16]. CellPhoneDB is a public repository consolidating information about receptors, ligands, and their interactions, as well as receptor complexes from scientific literature. Version 2.0 holds information on 978 unique proteins making up 113 complexes and 1397 receptor-ligand interactions. Data were extracted in June 2021 as XML file, and provided interactors of receptor-ligand interactions as well as receptor complexes were annotated with the Ensembl Gene ID. In our analysis, we focused on the subset of experimentally verified interactions and complexes holding in total 878 interactions. Joining this set of interactions with our lists of PD DEGs resulted in a set of receptor-ligand interactions and receptor complexes, with both interactors showing differential expression on the transcript level in at least one of the studies included in our meta-analysis. We subsequently evaluated the resulting list of interactions in the context of enriched GO biological processes. We also investigated how well the proteins of the receptor-ligand pairs are already studied in the context of PD. We therefore searched using the term “peritoneal” and the respective gene symbol in PubMed and manually inspected titles and abstracts of extracted publications for each of the receptors and ligands. Proteins without relevant studies in the context of PD were assigned into evidence category one because they were the most interesting ones. Proteins with one or two relevant studies in the context of PD were assigned to evidence category two. All other proteins holding three or more relevant publications in the context of PD were assigned to evidence category three. Emphasis in the Section 2 was placed on proteins from evidence category one.

### 3.5. Statistical Analysis and Data Manipulation

The statistical software R (version 4.0.2) was used for data management and data manipulation (https://www.r-project.org/, accessed on 1 October 2020). The following specific R packages have been used: UpsetR, limma, and GEO2R.

## 4. Conclusions

We consolidated transcriptomic data from 12 studies in the context of peritoneal dialysis and generated a set of 2591 differentially expressed genes that served as input to delineate dysregulated molecular pathways. We identified 70 receptor-ligand interactions, with both interactors being differentially in the context of PD. The receptor-ligand interactions BMPR2-BMP6, BMPR2-GDF6, FZD4-WNT7B, and ACKR2-CCL2, as well as the binding of EPGN and EREG to the EGFR, represent interesting connections for potential therapeutic intervention.

## Figures and Tables

**Table 1:** Transcriptomics studies included in our meta-analysis. PD = peritoneal dialysis; CKD5 = chronic kidney disease stage 5; wt = wild type; HPG = hyperbranched polyglycerol; and AlaGln = alanyl-glutamine.

Study Acronym	Platform	Sample Type	Group 1	Group 2	References
Zaza2008	Affymetrix U133A	patient PBMCs	PD	CKD5 (predialysis)	[6]
Reimold2013	Affymetrix U113 Plus PM	patient mesothelial cells	PD	Uremic	[7]
Scherer2013	Affymetrix U133 Plus 2.0	patient PBMCs	PD	CKD5 (predialysis)	[8]
Büchel2015	Affymetrix U133 Plus 2.0	primary mesothelial cells	PD fluids	control medium	[4]
Kokoroishi2016	Affymetrix U133 Plus 2.0	primary mesothelial cells	high glucose	normal glucose	[9]
Herzog2017	Illumina HiSeq 2000	patient peritoneal cells	PD without AlaGln	PD with AlaGln	[5]
Ruiz-Carpio2017	Agilent Whole Human Genome Microarrays Kit 4 × 44 K	patient mesothelial cells	epithelioid	non-epithelioid	[10]
Bartosova2018	Illumina Human Sentrix Beads	patient omental arterioles	PD	CKD5 (predialysis)	[11]
Han2019	Illumina HumanRef-8 v2.0	primary peritoneal cells	TGFB1 stimulated	wt	[12]
Liu2019	Agilent SurePrint G3 Human GE 8 × 60 K Microarray kit	mesothelial cell culture	without HPG	with HPG	[13]
Parikova2020	Illumina Human HT-12 v4 Expression BeadChips	patient peritoneal cells	long-term PD	short-term PD	[14]
Strippoli2020	GRCm38.76	primary mesothelial cells	stretched	non-stretched	[15]

**Table 2:** Top enriched GO biological processes. The numbers of DEGs assigned to the respective categories are provided next to the −log10 (adjusted *p*-value) in brackets for those categories showing significant enrichment. M = mesothelial cell DEGs; OA = omental arteriole DEGs, and P = peritoneal cell DEGs; PBMC DEGs.

GO Biological Process	All	M	OA	P	PBMC
angiogenesis	64 (4.59)	56 (3.53)	2	8	-
cell adhesion	110 (4.59)	88 (1.84)	-	23 (2.97)	3
positive regulation of cell migration	56 (4.59)	49 (3.53)	-	9	-
positive regulation of smooth muscle cell proliferation	28 (4.59)	23 (3.32)	3	4	-
inflammatory response	92 (3.99)	71 (1.33)	-	14	7 (5.66)
positive regulation of cell proliferation	107 (3.84)	90 (2.2)	9	12	1
extracellular matrix organization	55 (3.46)	47 (2.39)	-	12 (1.4)	-
positive regulation of angiogenesis	38 (3.46)	33 (2.66)	2	3	1
cell migration	49 (3.1)	39 (1.47)	-	10	-
leukocyte migration	38 (2.85)	31 (1.62)	4	4	1
response to estradiol	30 (2.28)	25 (1.54)	1	3	1
positive regulation of ERK1 and ERK2 cascade	47 (2.25)	37	2	9	-
cell division	79 (2.2)	76 (2.9)	1	2	-
cell-cell signaling	61 (2.04)	52	2	8	1
response to drug	70 (2.04)	60 (1.41)	4	8	1
positive regulation of MAP kinase activity	22 (2.03)	17	1	4	-
receptor internalization	18 (1.96)	17 (1.83)	-	-	1
regulation of phosphatidylinositol 3-kinase signaling	26 (1.93)	20	-	7	-
phosphatidylinositol phosphorylation	29 (1.76)	25	-	4	-
osteoblast differentiation	31 (1.76)	27 (1.46)	1	4	-
regulation of apoptotic process	52 (1.76)	46 (1.5)	1	4	1
protein autophosphorylation	44 (1.67)	39 (1.31)	-	5	-
cell-cell adhesion	62 (1.65)	54	6	2	-
positive regulation of tyrosine phosphorylation of Stat3 protein	16 (1.64)	15 (1.84)	1	-	-
response to hydrogen peroxide	19 (1.59)	16	-	3	-
positive regulation of vascular endothelial growth factor production	13 (1.57)	11 (1.33)	2	-	-
response to nutrient	24 (1.57)	21	-	3	-
negative regulation of apoptotic process	93 (1.56)	77	9	10	1
cellular response to retinoic acid	23 (1.55)	20 (1.31)	2	1	-
positive regulation of protein kinase B signaling	26 (1.55)	23 (1.38)	-	3	-
wound healing	25 (1.51)	19	2	5	1
response to ethanol	30 (1.47)	27	2	2	1
extracellular matrix disassembly	24 (1.46)	22	3	-	-
positive regulation of MAPK cascade	25 (1.46)	21	1	3	-
positive regulation of fibroblast proliferation	19 (1.38)	13	3	4	-
sprouting angiogenesis	12 (1.36)	12 (1.51)	-	2	-
mitotic nuclear division	56 (1.35)	54 (1.76)	1	1	-
collagen catabolic process	21 (1.33)	19 (1.34)	1	2	-
platelet degranulation	29 (1.33)	28 (1.76)	4	-	-
complement activation, alternative pathway	7	5	3 (1.85)	-	-
Rho protein signal transduction	15	11	4 (1.67)	-	-

## Data Availability

The set of consolidated differentially expressed transcripts can be accessed from the authors upon reasonable request.
